# Building community-engaged research capacity through bidirectional collaboration: the community research consulting initiative model at NYU Langone health

**DOI:** 10.3389/fpubh.2026.1816942

**Published:** 2026-08-18

**Authors:** Deborah Onakomaiya, Miriam Gofine, Michelle Chau, Kathleen Hopkins, Marilyn Fraser, Antoinette Schoenthaler, Nadia Islam

**Affiliations:** 1 Vilcek Institute of Graduate Biomedical Sciences, NYU Grossman School of Medicine, NYU Langone Health, New York, NY, United States; 2 Department of Population Health, NYU Grossman School of Medicine, NYU Langone Health, New York, NY, United States; 3 Institute for Excellence in Health Equity, NYU Grossman School of Medicine, NYU Langone Health, New York, NY, United States; 4 Department of Community-Based Programs, Family Health Centers, NYU Langone Health, New York, NY, United States; 5 Arthur Ashe Institute for Urban Health, New York, NY, United States

**Keywords:** academic-community partnerships, bidirectional learning, capacity building, community-based organizations, community-engaged research, research consultation

## Abstract

**Objectives:**

To develop and evaluate the Community Research Consulting Initiative (CRCI), a no-cost, student-led model designed to build bidirectional capacity between academic institutions and community-based organizations (CBOs) through research consulting and project-based assistance.

**Study design:**

This formative program evaluation examined the feasibility, acceptability, and preliminary outcomes of CRCI, which includes virtual Office Hours for short-term research consultation and Project-Based Assistance (PBA) for longer-term collaborations. The goal was to support CBOs with technical research needs while providing academic trainees with real-world experience.

**Methods:**

Academic consultants (students, staff, faculty) were recruited through institutional listservs and outreach and completed an intake form outlining their expertise and learning goals. CBOs were engaged through Community Advisory Boards, faculty referrals, and existing networks. Consultants provided support via Zoom-based Office Hours and were matched with CBOs for PBA through a memorandum of understanding. Data collection included Zoom registration logs, post-session surveys, and follow-up communications. Quantitative data were analyzed descriptively, and qualitative feedback was reviewed thematically to inform implementation improvements.

**Results:**

Thirty-three consultants expressed interest; 21 participated in Office Hours or PBA projects, representing a range of NYU departments. Consultants sought practical experience applying academic skills in community settings. Fifty-one unique CBOs registered for 90 Office Hours slots, primarily serving racial/ethnic minority and at-risk populations in New York City. Grant writing and data analysis were the most frequently requested topics. Among 20 completed surveys, 95% of CBO respondents found sessions useful and relevant, citing take-home resources as especially beneficial. Six CBOs engaged in PBA projects involving survey design, data analysis, and program evaluation. Consultants appreciated the learning opportunity but highlighted challenges related to attendance, preparation time, and expectation setting.

**Conclusion:**

CRCI demonstrates a feasible and replicable model for advancing equitable academic–community partnerships aligned with NIH priorities. The initiative supports community capacity building while fostering practical training for future leaders in community-engaged research. Broader implementation of CRCI-like models may enhance the sustainability and impact of academic–community collaborations.

## Background

The National Institutes of Health (NIH) calls for equitable academic and community partnerships (1). Collaborative research partnerships between academic institutions and local communities, including community-based organizations (CBOs), offer a valuable form of scholarship and a transformative approach to teaching and learning (2). CBOs are public or private nonprofit organizations representative of a community or significant segments of a community and provide educational or related services to individuals within the community (3, 4). CBOs are experts in the assets and needs of the local communities they serve, but often face challenges related to workforce capacity, funding, access to technologies, and human resources (5). Academic faculty, students, and staff, on the other hand, may be disconnected from real-world research practice. For example, while many possess the necessary technical and theoretical knowledge, they often struggle to apply these skills effectively, adapt quickly, and navigate real-world challenges, highlighting the need for hands-on experiences (6). This disconnect can present a problem for both CBO and academic institutions because academic researchers may not have insight into communal priorities, needs, and concerns. Experiential learning in community settings can equip students and researchers with hands-on skills and real-world exposure, ensuring they are career-ready for work in practical or applied settings (7, 8). At the same time, community-academic research partnerships can build capacity for CBOs that may not otherwise have access to academic resources, staff, and research support (3).

Community-academic partnerships offer unique opportunities to draw from the respective strengths and expertise of academic institutions and community partners to achieve health equity, especially among socially at-risk communities (9, 10). These partnerships can help promote high-quality research and capacity building through the creation of systems to enable the translation of research findings into action to improve health equity; support new knowledge to inform policy and decision-making; implement and sustain innovative interventions, critically analyze existing systems of health service delivery, governance structures, and social determinants of health, and pool resources to better address health inequity (9, 11). Therefore, there is a need to invest in supporting collaborative academic and community partnerships.

Despite the many advantages of community-academic partnerships in bridging the gap between academic institutions and CBOs, they often face persistent challenges that inhibit their effectiveness and durability. Many partnerships struggle with team stability, as frequent personnel changes disrupt research progress and weaken trust (12, 13). Communication and logistical barriers, including challenges coordinating schedules and keeping partners informed, further hinder collaboration. Additionally, CBOs often operate with limited resources, making it difficult to fully engage in research efforts (12, 13). While Memorandums of Understanding (MoUs) attempt to formalize partnerships, they often fail to ensure long-term commitment and equitable resource distribution (14, 15). Institutional barriers, such as rigid academic policies on funding and research leadership, can further restrict community participation (13). Sustainability remains a major concern, as many initiatives lose momentum after initial funding cycles (13). Differences in organizational structures, priorities, and populations served can also create misalignment in expectations and research objectives (13).

Designed to directly address key challenges such as unstable collaboration, limited communication, and inequitable access to expertise, we developed a Community Research Consulting Initiative (CRCI). The CRCI offers a structured, no-cost model of bidirectional capacity building between academic and community-based partners to create intentional pathways for sustained partnership. Through matching community partners with academically trained research consultants from diverse academic backgrounds, CRCI promotes mutual learning, fosters trust, and enhances the long-term effectiveness of research collaborations. This innovative approach strengthens both academic and community capacity, offering a potentially sustainable solution to common barriers in community-academic partnerships.

CRCI distinguishes itself through its focus on structured experiential training for trainees, early-stage investigators, and staff who might otherwise lack formalized opportunities. While community-engaged research (CEnR) training programs have long emphasized co-learning and reciprocal knowledge exchange, many lack the consistent structure needed for long-term impact (16). Capacity-building frameworks in CEnR often prioritize experiential learning and leadership development, both of which are essential for research partnerships (17). CRCI addresses this gap by offering a more robust and structured training model that focuses on skill development through immersive, real-world research experiences. By doing so, CRCI not only enhances research capacity among academic investigators and community partners but also fosters leadership in community-engaged research, building on and expanding the foundations set by previous community-engaged training programs.

Conceived of and led by two doctoral students in the Institute for Excellence in Health Equity & Department of Population Health (DPH) within NYU Grossman School of Medicine (NYUGSoM), CRCI facilitated collaboration between CBO staff and volunteer students, staff, and faculty as research consultants across a large health system. We present the process of CRCI’s development, implementation, and lessons learned, outlining the process of developing a community-focused academic initiative informed by feedback from diverse partners across academic and community settings alike.

### Development of CRCI

Our volunteer work with CBOs informed the concept of CRCI for bidirectional capacity building, shaped by a shared recognition of two pressing needs:

1. The need for academic trainees to gain a deeper understanding of the research priorities and realities within community settings, and
2. The need for many CBOs to build and strengthen capacity in areas aligned with the rigorous research skills fostered through academic training.

What began as a simple idea to create opportunities for semi-formal skill exchanges between advanced trainees with experience in human subject research and CBO partners with occasional research needs gradually evolved into the structured CRCI model. A formal charter was not developed for CRCI. However, the founders established a guiding mission and set of objectives: to build bidirectional research capacity by connecting CBOs with academic research consultants in a no-cost, reciprocal model that strengthens community research capacity while providing academic trainees with applied, community-based experience. These mission and objectives were developed by the two doctoral student co-founders in consultation with senior faculty advisors (NI and AS) and were refined iteratively through the stakeholder feedback described above. Although not codified in a single charter document, the mission and objectives were consistently embedded throughout CRCI’s recruitment materials, orientation, and partner-facing communications.

We envisioned that consultants would volunteer their expertise in various areas, including data analysis, collection, and management; survey development; and language translations, among others, to CBOs that have research needs but lack the necessary expertise and/or time and bandwidth to conduct these activities internally. In return, CBO partners would offer research consultants valuable experiences with applied research within community settings and an enhanced understanding of their CBO’s role, mission, and strategies used to advance community priorities. This experience would include learning about partner engagement and sustainable partnership building. From the community’s perspective, this model’s collaborative framework would enhance the problem-solving capacity of CBOs and direct research efforts toward addressing critical needs identified by the community itself.

To actualize our idea, we approached senior faculty advisors who are experts in community-academic partnerships (NI and AS) in August 2021. We subsequently met with faculty, post-doctoral fellows, and staff over a 4-month period (August 2021 to January 2022) to refine and solicit feedback on the concept. This phase was conducted through a series of online meetings in which we presented a general overview of the proposed program and invited qualitative feedback on its structure and design. Feedback was gathered conversationally rather than through formal surveys. We intentionally sought input from individuals representing both sides of the proposed exchange: those who would primarily give and teach (e.g., staff and post-doctoral fellows) and those who would primarily receive support (e.g., CBO-based partners), as well as what each group was willing to contribute. As we moved from one group to the next, we incorporated and carried forward earlier suggestions, presenting each subsequent group with the adaptations prior groups had proposed and asking for their reactions; feedback was then aggregated across groups. This iterative, developmental phase was distinct from the later structured stakeholder feedback described below, which focused on refining specific implementation features and evaluation mechanisms. Following this phase, we solicited community feedback on CRCI’s model and implementation strategy from an established Community Advisory Board (CAB) affiliated with the NYU-Health + Hospital Clinical and Translational Science Institute (spring 2022). The CAB consists of leaders from the community, healthcare, and academic/research sectors who guide strategic development, facilitate partner engagement, and provide critical feedback. Their primary mission is to create healthier communities across New York City by fostering equitable research partnerships between community partners and researchers. CAB members play a key role in shaping community-engaged research strategies, identifying research priorities, and offering a community perspective on the development, implementation, and dissemination of research and research training programs at NYUGSoM. By leveraging institutional resources and collaborating with community health organizations, they help translate prevention research into sustainable practices. This partnership ensures that initiatives like the CRCI model effectively address community needs and drive meaningful health improvements. As a final step, we also solicited feedback on administrative intake material by directly sharing it with individual CBO-based partners. CBO-based partners were engaged via email, and we shared materials with them for feedback. CBOs we reached out to were focused on serving at-risk youth across NYC.

### Preliminary feedback of the CRCI structure and process

We also sought input from a range of stakeholders, including NYU Langone faculty (NI, AS, CB), students, CBOs, and members of our CAB. Using a formative, iterative engagement approach, we conducted informal interviews and structured feedback sessions to understand priorities related to community-academic partnerships, engagement best practices, and structural supports for research collaboration. Faculty emphasized the need for structured feedback mechanisms, which led us to incorporate post-office hour surveys and follow-up interviews with consultants. Students shared perspectives on fostering trust and reciprocity in community partnerships, reinforcing the importance of bidirectional learning with CBOs.

In our outreach to CBOs, we prioritized organizations aligned with the research expertise and ideals of CRCI. For example, we engaged a youth-serving CBO identified through NYU’s community engagement networks because of their experience supporting historically underserved populations and their potential alignment with CRCI’s mission. We invited this CBO to review early CRCI materials via email to ensure accessibility and relevance from the perspective of grassroots organizations. Their feedback emphasized the value of focused, topic-specific sessions and helped us tailor office hours to better meet community needs.

Table 1 summarizes the stakeholder groups we engaged, the nature of input provided, and the resulting adaptations to the CRCI model.

**Table 1:** Partner insights and actionable outputs for developing the community research consulting initiative (CRCI).

Advisor group	Areas of guidance	Our action
NYU Langone Faculty Advisors	CRCI evaluation strategy	Implement post-office hours evaluation surveys Conduct check in interviews with consultants
Post-doctoral students engaged in community-based research	Best practices in community-engaged research and associated programs	Maintain strong focus on bidirectional learning with CBOs
CBO Partners, NYU CAB and NYU departmental Staff engaged in community-based research	Structured formats for Office Hours	Maximize engagement and learning during office hour’s sessions by centering them on focused, topic-specific discussions.

Informed by academic and community partner feedback, CRCI’s concept was solidified into 2 formats:

1. 1 *Office Hours*: We conceptualized Office Hours as one-hour drop-in sessions focused on a specific topic. This model allowed consultants to provide real-time assistance to CBO partners on particular topics during a brief session, without requiring any long-term commitment from CBO or consultant participants. Consultants were not expected to prepare a lecture or teaching materials. Our faculty advisors recommended maintaining a singular focus for each *Office Hours* session, allowing consultants to highlight their expertise effectively and ensuring that sessions remain focused. Office Hours were offered in sets of approximately 8-week sessions, beginning with a pilot set of six sessions. Within each set, individual sessions were not held on a fixed weekly basis; rather, single sessions were scheduled at intervals of approximately one to 2 weeks across the block, with only one session offered at a time (no concurrent or multiple weekly sessions). The specific dates, times, and topics for each set were pre-scheduled and published in advance on a publicity flyer once consultant hosts had confirmed their availability (see Appendix), rather than being added *ad hoc* as the block progressed. Topics and the number of sessions per set were planned in advance of each block and adjusted between blocks based on consultant availability and CBO feedback.
  *Office Hours* were hosted by 1–2 consultants with expertise in the given topic; attendees were encouraged to arrive with prepared questions. Following the session, we distributed evaluative surveys to consultants and CBO partners. Our evaluations emphasized the mutual benefit of the capacity-building opportunity, asking both academics and the community about what they offered and gained from the collaboration. Topics for office hours were selected based on our initial consultations with community members and consultant expertise. *Office Hours* meetings were hosted on Zoom.
2. 2 *Project-based assistance (PBA)*: This mode paired consultants and CBOs as longer-term partners to more formally facilitate bi-directional capacity building. This initiative offered CBOs sustained, focused assistance while simultaneously providing CRCI consultants with opportunities to learn and develop skills through their collaboration with CBOs. The two formats were intended to produce different outputs. The expected output of an Office Hours session was real-time, topic-specific consultation and the sharing of take-home resources within a single one-hour session, with no expectation that consultants prepare formal teaching materials and with attendees expected to arrive with prepared questions. The expected output of a PBA engagement was a defined, negotiated deliverable produced over a minimum three-month collaboration with regular check-ins, such as a survey or intake tool, a completed data analysis or visualization, or a program evaluation, as agreed in the memorandum of understanding.

## Methods

### Study design

This formative program evaluation examined the feasibility, acceptability, and preliminary outcomes of CRCI, which includes virtual Office Hours for short-term research consultation and Project-Based Assistance (PBA) for longer-term collaborations. The goal was to support CBOs with technical research needs while providing academic trainees with real-world experience. This manuscript was prepared in accordance with the Standards for Quality Improvement Reporting Excellence (SQUIRE 2.0) guidelines, and a completed SQUIRE 2.0 checklist is provided as a Supplementary file.

### Consultant recruitment and orientation

Student, staff, and faculty consultants from our department were periodically recruited through a listserv reaching all our department’s affiliates (approximately 500 recipients); presentations at departmental and division meetings (approximately 300 attendees); and Q&A Zoom-based recruitment sessions. Consultants across the institution were also recruited through a listserv that reached all master’s, doctoral, and postdoctoral students across the academic medical system (approximately 300 recipients) and engaged key individuals at schools across NYU, including the School of Social Work, College of Global Health, and Department of Sociology. We encouraged all recipients of our publicity emails to circulate the opportunities within their personal and professional networks.

Individuals who expressed interest in joining CRCI as consultants were required to complete an intake form, which collected information on their preferences for Office Hours and/or PBA. The form gathered details such as areas of expertise, language proficiency beyond English, availability, and their goals for learning from CBO partners. Following the submission of their forms, all consultants participated in a brief orientation session. See the intake form in the Appendix.

The orientation was designed primarily to promote consistency across sessions and to set expectations rather than to deliver formal training in community-engaged research; consultants were selected on the basis of existing research expertise relevant to their chosen topic, and no additional formal coursework, certification, or competency-based training was required prior to participation. During the orientation, consultants were encouraged to begin the session with a brief structured introduction of themselves and the attendees; we provided an optional PowerPoint template to guide this introduction. Consultants were aware that attendees were invited to come with prepared questions and were not required to independently prepare materials. Beyond the orientation, the guidance consultants received consisted of the optional introductory template and, for those participating in PBA, example MoUs and a standardized MoU template that were shared to structure their engagements (see Appendix). Consultants who decided to proceed with hosting one or more *Office Hours* sessions volunteered their time on an online schedule we posted with topic, time, and date. The implications of this orientation-based approach are addressed in the discussion.

### CBO recruitment

CBO partners were invited to participate in CRCI’s activities through multiple channels: presentations to two CABs; targeted outreach to CBOs by departmental faculty members already engaged in community-academic partnerships; inviting recipients of all publicity emails to invite colleagues at other CBOs; and outreach to personal CBO contacts. We used digital flyers to circulate information about the initiative and specific upcoming opportunities. Prospective CBO partners were invited to join a Mailchimp mailing list to be notified of CRCI opportunities.

### CBO-consultant matching

We began matching consultants and CBOs for longer-term projects in October 2022. Participants were required to commit to a minimum of 3 months and co-complete a standardized memorandum of understanding (MoU).

The CBO partner and CRCI consultant were introduced via email, and both parties were requested to complete and submit a structured MoU template. The MoU was intended to facilitate a discussion regarding collaboration expectations, including:

1. Project timeline: Establishing deadlines and milestones for project completion
2. Meeting parameters: Defining scheduling, frequency, and duration of meetings
3. Skill share agreement: Articulating the skills that both parties aim to share and learn during the collaboration
4. Potential academic outputs or on-site visits: Discussing the scope and parameters of any academic publications or on-site visits resulting from the collaboration

Regular check-ins were scheduled with consultants and CBOs to ensure the project’s smooth progress. A single standardized MoU template was used as the starting point for all PBA pairings (see Appendix); each MoU was then personalized to reflect the specific details of the engagement between the CBO and consultant, including the project focus, meeting cadence, anticipated duration, and the skills each party expected to give and receive. The template specified the anticipated start and end dates and noted that the consultancy could be re-evaluated for extension by all partners at the end date; in this sense, the agreed end date functioned as the principal exit parameter, and either party could decline to extend. The MoU also documented the consultant’s anticipated weekly time commitment and availability between meetings. Because CRCI consultants volunteered their time, the MoU was designed in part to make the scope and limits of that voluntary commitment explicit and mutually agreed; had any compensation been involved, the template provided for it to be stated, along with the associated number of hours. We did not encounter any situation involving the exploitation of unpaid academic labor, and the explicit, bounded commitments recorded in each MoU were intended to guard against this possibility. The anticipated contributions and expectations negotiated through these MoUs are summarized in Table 2.

**Table 2:** Summary of anticipated PBA CBO and consultant exchange expectations as reported in memoranda of understanding.

Project No. (Anticipated duration)	Giving		Receiving	
	CBO	Consultant	CBO	Consultant
1 *(3 months)*	Opportunities for involvement in grant-writing activities Assistance with data collection	Formative evaluation of CBO services Qualitative data collection Knowledge transfer to CBO leadership on implementing ongoing internal evaluation activities	Formative evaluation of CBO services	Professional development (Experience with grant-writing; applied evaluation experience in community setting)
2 *(4 months)*	Staff availability	Data analysis and synthesis Creating an evidence-based narrative for fostering policy change	Evidence-based narrative for fostering policy change	Applied research experience in community setting Access to CBO datasets Better understanding of population CBO serves
3 (4 months)	Experience and exposure to community engaged participatory research Exposure to CBOs multi-disciplinary approach to community engagement and research Access to internal datasets Deeper understanding of community needs	Quantitative data analysis Drafting data visualizations, manuscripts, reports and/or abstracts Identifying areas for analyses	Technical assistance in establishing a framework for analyzing data Support analyzing qualitative and quantitative data and identifying opportunities for publication Drafted data visualizations, manuscripts, reports and/or abstracts	Applied research experience in community setting (types of and reasons for data collection, uses of analyzed data, involvement of different partners in data analysis process) Better understanding of population served by CBO
4 (5 months)	Access to internal documentation system Staff availability	Expertise and brainstorming regarding program evaluation Resources Review of documents, procedures	Expertise in conducting evaluation of CBOs mission and vision	Access to documents; regular communication
5 (10 months)	Staff availability to provide qualitative and quantitative data Collaboration on development of survey tool and analyzing results	Survey for needs assessment of CBOs target population Training session on data collection for program staff Bi-weekly data analysis discussion sessions with program staff over 10 month period	Survey for needs assessment of CBOs target population Training sessions data collection and analysis	Contextual knowledge of CBOs goals and background Collaboration on development of survey tool and analyzing results
6 (12 months)	Sharing experience from a community perspective Data created in the research project	Consulting for overall research project (e.g., study design, survey development, human subjects protections)	Consulting regarding research project Preparation for IRB submissions	Data shared in the research project Authorship of any academic outputs from the collaboration

### Data collection

#### CBO office hours registration

CBO partners registered their names, affiliations, demographics, how they learned about CRCI, and any questions for consultants through a survey created on the Zoom platform. Additionally, an exit survey was distributed to attendees after the session to gather feedback. However, due to limitations of the Zoom platform, we were unable to track the exact number of attendees per session. A pre-registration link was used to capture the number of registrants per session, and an exit survey was used to gather attendee feedback; however, sessions were not recorded, as recording would have required obtaining additional consent from attendees. Consequently, we relied on consultant-reported attendance counts rather than verified counts from a recording, which we note as a limitation.

#### Office hours evaluation

Office hours evaluations were conducted using separate surveys of CBO attendees and consultants. Responses to both surveys were collected anonymously using the Qualtrics platform. Consultants distributed the URL for the CBO surveys to attendees in the last few minutes of the session. We followed up with consultants after each session by directly asking how the session went in a follow-up email and including the consultant survey URL. Surveys were very brief to minimize respondent burden and increase the likelihood of response.

CBO surveys asked respondents to report which populations(s) they worked with; provide free-text responses to evaluations about session strengths and areas for improvement; and rate their agreement with the following five statements on a 5-point Likert scale:

1. “I learned something useful from today’s consultation”
2. “The consultant(s) was knowledgeable about the training topic”
3. The consultant(s) answered questions adequately
4. “The information I received from today’s consultation was helpful”
5. “I am likely to utilize the information I received”

Respondents were invited to suggest topics for future sessions.

Consultant surveys asked consultants to report the number of attendees; to rate their confidence in their ability to respond to attendee questions on a 5-point Likert scale; and provide free text responses to evaluate the communication from CRCI leaders (DO and MG); how the session went; what they learned from the session and how hosting the session would influence their future work.

#### Project-based assistance evaluation

Qualitative evaluative feedback was requested from CBO partners and consultants 6 to 8 weeks after initially introducing consultants and partners. We limited solicitation of feedback to a brief email (“Checking in – how is it going?”) to minimize respondent burden and increase the likelihood of receiving a response. We also planned to use the information we received to develop a more formal evaluation once our knowledge was more robust. Based on guidance from the NYU Grossman School of Medicine Institutional Review Board (IRB), this program was determined to be exempt, as it was conducted as a quality improvement initiative rather than human subjects research.

### Analysis

We used a primarily descriptive approach to analyze both quantitative and qualitative data collected from CBO Office Hours and PBA surveys and email follow-ups. Data were analyzed retrospectively, after data collection across the Office Hours sets and PBA engagements had concluded, rather than on a continuous or pre-scheduled interim basis during implementation.

Quantitative survey responses from CBO partners and consultants were summarized using basic descriptive statistics, including frequency counts and means for Likert-scale items. This included metrics such as the percentage of CBO participants who agreed or strongly agreed with statements about the usefulness and relevance of the consultations, and consultant self-ratings of confidence in responding to questions.

For qualitative data, including open-ended survey responses and email feedback, we conducted a basic content review to identify common themes. Responses were reviewed for recurring ideas related to session satisfaction, consultant helpfulness, and suggestions for improvement. These themes were used to inform ongoing program adjustments. However, no formal coding or in-depth thematic analysis was conducted, in keeping with the program’s quality improvement focus. This straightforward analytic approach was employed to monitor trends, gather practical feedback, and make timely, data-informed decisions to strengthen CRCI implementation.

## Results

### Consultant characteristics

**Figure 1:**
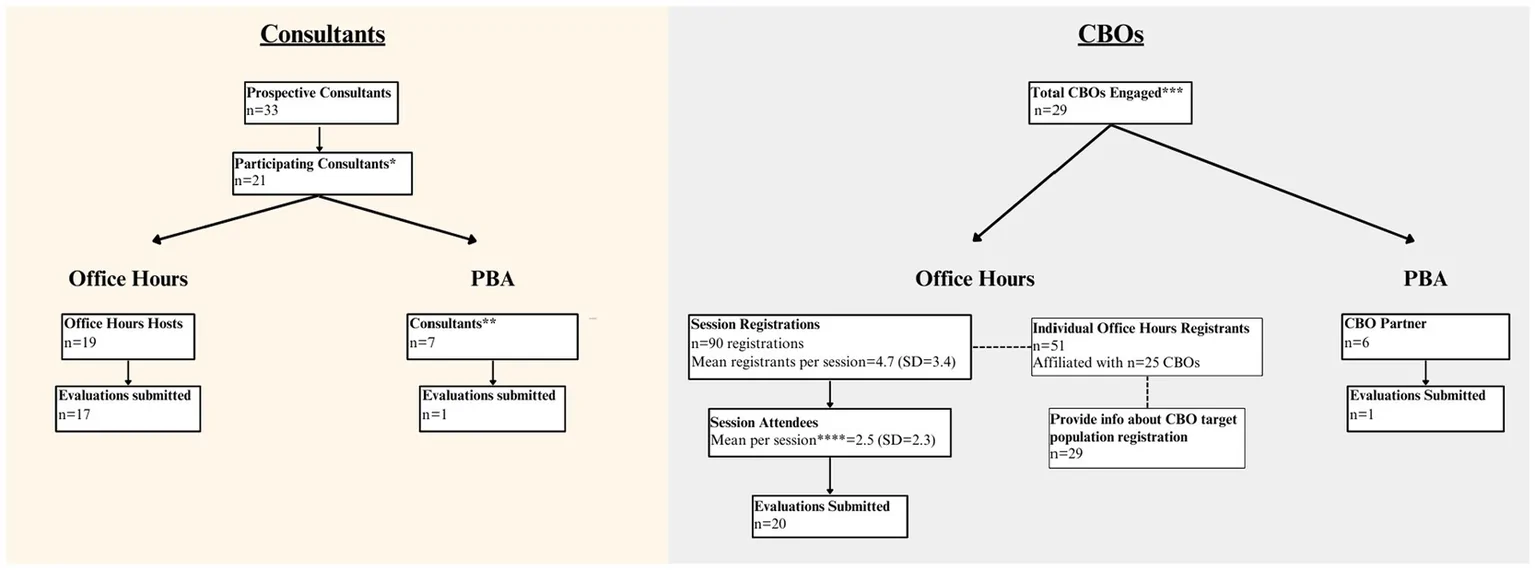
Modified CONSORT diagram.*4 Consultants both hosted Office Hours and participated in PBA; 2 PBA consultants did not participate in Office Hours.**1 PBA consultant withdrew mid-match and was substituted with another consultant.***22 CBOs participated in Office Hours only; 3 participated in PBA and Office Hours, 3 participated in PBA only.****As reported by consultants in 17 post-session evaluations.

In total, 33 prospective consultants indicated interest in hosting *Office Hours* and/or PBA. As detailed in Figure 1 and Table 3, 21 consultants ultimately hosted at least one *Office Hour* and/or engaged in one PBA partnership. Consultants were affiliated with multidisciplinary entities across NYU schools and departments, including NYUGSoM, NYU Silver School of Social Work, NYU Elmer Holmes Bobst Library and NYU Graduate School of Arts & Science. Of these 21 consultants, 19 hosted at least one Office Hours session and 6 participated in a PBA engagement; 4 consultants participated in both formats, and 2 consultants participated in PBA only (see Figure 1).

**Table 3:** Implementation of office hours (Fall 2022-Spring 2023).

Type	Characteristics	*n* (%)
Consultants	Total office hours sessions	19
	Average hosts per session	1.5 (SD 0.5)
	Consultants, by type	21
	Doctoral student	12 (57.1%)
	Faculty	2 (9.5%)
	Postdoctoral student	1 (4.8%)
	Research Staff	6 (28.6%)
	Hosting frequency^†^	
	1 session	11 (57.9%)
	2 sessions	7 (36.8%)
	3 sessions	1 (5.3%)
	Skills^‡^	
	Data analysis (quantitative)	12 (57.1%)
	Data analysis (qualitative)	8 (38.1%)
	Study design	12 (57.1%)
	Data collection^¶^	12 (57.1%)
	Survey development	10 (47.6%)
	Program evaluation	7 (33.3%)
	Grant writing	6 (28.6%)
	Educational material development	6 (28.6%)
	Communications/marketing^††^	4 (19.0%)
	Other^‡‡^	4 (19.0%)
	Non-English language fluencies	
	Hebrew	1 (4.8%)
	Hindi	1 (4.8%)
	Korean	1 (4.8%)
	Portuguese	2 (9.5%)
	Spanish	5 (23.8%)
CBO Partners	Total registrations across all Office Hours^¶¶^	90
	Unique registrants, by type	51
	Affiliated with non-profit CBOs	21 (41.2%)
	Affiliates of other universities and academic medical centers	4 (7.8%)
	Departmental post-docs, faculty and research staff	22 (43.1%)
	Clergy	2 (3.9%)
	Employees of for-profit business	2 (3.9%)
	Session registration frequency	
	Registered for 1 session	32 (62.7%)
	Registered for 2 sessions	9 (17.6%)
	Registered for 3 sessions	10 (19.6%)
	Average # of registrants	4.7 (SD 3.4)

^†^4 consultants both hosted Office Hours and participated in PBA; 2 PBA consultants did not participate in Office Hours; 1 PBA consultant withdrew mid-match and was substituted with another consultant. Office Hours sessions were hosted by a minimum of one and a maximum of two consultants. ^‡^Sums may exceed 100% due to multiple selections. ^¶^e.g., Focus groups, interviews, intake forms. ^††^e.g., Canva, MailChimp, social media. ^‡‡^Data visualization; poster/infographic design; environmental justice issues; public/environmental health curriculum for CBOs; integrating data-based decision making; thought partnership; workflows utilizing publicly accessible resources. ^¶¶^This number includes multiple registrations per person. Session attendance data are unavailable due to Zoom platform limitations. Denominators for reported percentages: Consultant “by type,” “Skills,” and “Non-English language fluencies” rows are calculated out of the 21 consultants who participated in Office Hours and/or PBA (*n* = 21). The “Hosting frequency” rows (†) are calculated out of the 19 consultants who hosted at least one Office Hours session (*n* = 19); because some sessions were co-hosted, the 19 Office Hours hosts delivered a total of 19 sessions, with an average of 1.5 hosts per session. CBO Partner “Unique registrants, by type” and “Session registration frequency” rows are calculated out of the 51 unique CBO registrants (*n* = 51); “Total registrations across all Office Hours” (¶¶) reflects 90 total registrations and includes multiple registrations per person. Percentages for “Skills” (‡) may exceed 100% because consultants could select multiple skills.

Upon sign-up, consultants were asked to describe in their own words what skills and experiences they hoped to gain from CBO partners. Consultants commonly noted interest in strengthening community partner engagement skills; field experience in using academic training in applied settings; better understanding of CBO data needs and the data that CBOs generate.

Table 3 describes the diverse skillsets of the consultants, including their language fluencies.

### CBO characteristics

As detailed in Figure 1, 51 unique CBO registrants signed up for a total of 90 registrations for Office Hours: 32/51 registrants (62.7%) signed up for one Office Hours session; nine (9/51, 17.6%) registered for two sessions; and 10 (10/51, 19.6%) registered for three or more sessions.

As detailed in Table 3, while 21 (21/51, 41.2%) registrants were affiliated with non-profit CBOs, we unexpectedly observed registration by individuals not affiliated with a variety of external entities, including faith-based organizations and other academic medical centers. Job titles of the 21 attendees affiliated with non-profit CBOs indicated a variety of roles, including a patient navigator (*n* = 1/21, 4.8%), interns (*n* = 2/21, 9.5%) and individuals with associate or coordinator-level titles (*n* = 7/21, 33.3%), manager-level titles (*n* = 2/21, 9.5%), and director-level (*n* = 9/21, 42.9%) titles.

Twenty-nine registrants (29/51, 56.9%) completed an optional registration field requesting a description of the target population(s) served by their CBO. These CBOs were New York City-based and focused their efforts on various racial/ethnic minority, immigrant and at-risk populations.

The six CBOs participating in PBA were also based in New York City. One focuses on at-risk LGBTQ+ youth; one focuses on supporting populations with long COVID; two focus on serving specific immigrant communities; and two conduct grassroots organizing for social justice-related causes. Implementation of Office Hours and PBA is described in Table 3.

### Office hours

#### Reach and tracking of office hours

Office Hours began with 6 pilot sessions from June to August 2022, followed by 6 sessions in November 2022 to February 2023, and 7 sessions in June to August 2023.

Office Hours topics included: grant writing; qualitative and quantitative data analysis (as separate sessions); data collection; program evaluation; survey development and data management (focusing on free or low-cost platforms such as Excel, Google Forms, and Survey Monkey); data visualization; literature reviews; and application of mixed methods.

#### CBO survey results

Of 20 submitted Office Hours CBO session evaluations, 19 (95%) agreed or strongly agreed with each of the following statements: “I learned something useful from today’s consultation”; “The consultant(s) was knowledgeable about the training topic”; “The information I received from today’s consultation was helpful”; “I am likely to utilize the information I received.” The full CBO and consultant survey instruments are provided in the Appendix.

Among the 12 (60%) of CBO respondents who provided free-text responses to the question, “What was useful about today’s consultation?,” many attendees emphasized the usefulness of take-home resources, particularly those related to grant writing. In the words of three attendees:

*Attendee 1 “A few resources I can use right away”.*
*Attendee 2 “I received a lot of resources to refer to in my daily work”.*
*Attendee 3 “[The consultant] broke down what grant writing is and later shared with me free/ accessible resources to further my journey into grant writing!”*

A desire for takeaway resources, particularly on grant writing, was strongly expressed by attendees in response to post-session evaluations. Interestingly, while only 6 (6/21, 28.6%) consultants indicated expertise in grant writing, sessions on grant writing were among the most popular and enthusiastically received by attendees, as reported in post-session CBO evaluations and anecdotally by hosts. In response to CBO feedback, we increased the frequency of Office Hours sessions offered on grant writing to twice instead of the typical once per publicized set of sessions.

#### Consultant survey feedback

1 Office hours session structure

Pilot evaluations indicated that some CBO partners attended without a specific question or concern and appeared to expect a more structured lecture or seminar format. As we intentionally did not require consultants to prepare materials to minimize consultant time burden, this resulted in less efficient use of session time for consultants and attendees alike.

In response, we revised our marketing materials to more clearly promote the sessions as “Q&A” events, encouraging attendees to come prepared with a list of questions. We also updated our registration process, asking CBO partners to submit their questions during session sign-up.

Despite these changes, post-pilot evaluations continued to reveal that many CBO partners arrived without specific questions or concerns,

*OH Consultant 1 “Some [attendees] wanted a short presentation/list of resources. In the future, it might be nice to have presenters pull together a few slides introducing the topic and provide links to helpful references that folks can take away from it. People had questions, but they more were expecting a lecture/overview of program evaluation.”*

*OH Consultant 2 “I wonder if participants thought the session was more of a webinar/lecture?”*

Paralleling this, some post-pilot CBO attendees requested a more structured session and take-home materials in response to the evaluation question,” What could be improved [about today’s session]?”:

*Attendee 4 “a take away pdf of contact info and links shared during the discussion.”*

*Attendee 5 “more structure to sessions”*

3 Low office hours attendance

On average, 4.7 (SD 3.4) people registered per Office Hours session, yet consultant hosts reported an average of 2.5 attendees per session (SD = 2.3) in post-session evaluations. Consultants shared concerns about no or low attendance at Office Hours sessions, expressing that it may have impacted their motivation to remain engaged with the CRCI initiative. To address this, we amended our consultant orientation materials to emphasize that low or no attendance was a possibility.

1. Low Response to Office Hours Evaluations

Despite our best efforts to collect CBO evaluations at the end of each session, we were challenged to achieve high response rates. Session evaluations were submitted by only 20 attendees.

### Project-based assistance

#### Implementation

Invitations were sent to CBOs via email, inviting them to submit proposals for PBA. Six CBOs expressed interest in collaborating with consultants on various projects, spanning topics such as program evaluation and needs assessments. After reviewing the proposals, we facilitated the matching process by providing CBOs with an anonymized list of five appropriate consultants along with details of their expertise, based on a match between consultant and CBO skills and interests. CBOs were then asked to provide feedback and rank their top three preferences. Subsequently, consultants were contacted in the order of CBO partner preference to confirm their availability.

Table 2 summarizes the mutual expectations between consultants and CBOs, detailing contributions and support for each project. Three consultants revised data collection tools, like surveys and intake forms. One conducted quantitative analyses and created data visualizations from a CBO’s health assessment data. Another evaluated a CBO’s programs to enhance recruitment and retention strategies. One partnership did not progress beyond an initial meeting due to competing priorities and scheduling conflicts. This highlights both successful collaborations and challenges faced across the PBA engagements. Table 4 summarizes the six PBA projects in greater detail, including the anticipated duration of each engagement, the nature of the project, the documented outputs, and IRB requirements. None of the six PBA projects required separate IRB review, as each was conducted as a program-improvement or capacity-building activity with the partnering CBO; no formal academic outputs (e.g., peer-reviewed publications or conference posters) were documented during the evaluation period, although several engagements produced practical deliverables such as revised surveys, intake tools, data analyses, and program-evaluation materials for the CBO partners. Several patterns distinguished engagements that progressed well from those that did not, which may help readers anticipate when this model is most useful. Engagements that worked well tended to involve a concrete, well-scoped deliverable that matched the consultant’s existing expertise and the CBO’s immediate need, together with a CBO partner who had identified data or materials ready to work with and who maintained regular communication. For example, one engagement produced quantitative analyses and data visualizations from a CBO’s existing health-assessment data, another delivered a formative program evaluation with recommendations for volunteer training and retention, and others revised surveys and intake tools that the CBO could continue to use; one CBO partner described the beginning of a sustained collaborative relationship extending beyond the formal engagement. By contrast, the engagement that did not progress beyond an initial meeting was characterized by competing organizational priorities and scheduling conflicts that prevented the partnership from establishing a shared, actionable scope. Consultants also identified recurring friction points even within successful engagements, including the time commitment required, delays in partner responsiveness when intermediate work needed feedback, and the challenge of aligning expectations about roles, as reflected in one consultant’s account of navigating a perceived power dynamic in which the consultant was viewed as the academic “expert.” Taken together, these examples suggest that PBA is most likely to succeed when partners can agree early on a focused deliverable, confirm that the necessary data or materials and staff availability are in place, and establish a realistic, mutually owned timeline; conversely, pairings without a clearly bounded scope or sufficient partner bandwidth may benefit from beginning with a lower-commitment Office Hours interaction before committing to a longer-term project.

**Table 4:** Summary of the six Project-Based Assistance (PBA) engagements.

PBA	CBO focus area	Anticipated duration	Project focus/activities	Documented output	IRB review required
CBO 1	Urban health / community health transformation	~4 months	Quantitative analysis and data visualization of CBO health-assessment data	Data analyses and visualizations for CBO; no formal academic output documented	No
CBO 2	Long COVID support	~4 months	Formative evaluation of community education, navigation, and translation services; volunteer training and retention	Evaluation memo(s) for CBO leadership; no formal academic output documented	No
CBO 3	Affordable housing / social and racial justice	~4 months	Data synthesis and development of a research-backed narrative to support housing policy change	Synthesis/narrative materials for CBO; no formal academic output documented	No
CBO 4	LGBTQ+ youth (faith-based community focus)	~5 months	Program evaluation and revision of intake forms and data-collection processes	Revised intake and data-collection tools for CBO; no formal academic output documented	No
CBO 5	Immigrant community services (Korean American)	~12 months	Consulting on overall research project: research design, survey design, and IRB application support	Study design and survey materials; potential future authorship discussed; no formal academic output documented during evaluation period	No (CBO-led IRB anticipated, separate from CRCI)
CBO 6	Immigrant community services (South Asian / multi-ethnic)	~10 months (extended from initial ~3-month term)	Development of a health and social needs assessment survey and staff training on data collection and analysis	Needs-assessment survey and staff training for CBO; manuscript discussed but contingent on future IRB approval; no formal academic output documented during evaluation period	No (any future manuscript contingent on separate IRB approval)

CBOs are de-identified (CBO 1–6) to protect partner confidentiality. Durations reflect the anticipated start and end dates recorded in each memorandum of understanding (MoU). One additional consultant-CBO pairing did not progress beyond an initial meeting due to competing priorities and scheduling conflicts and is therefore not represented as a completed engagement above. No PBA project required separate IRB review; potential future manuscripts noted for two engagements would require separate IRB approval before being undertaken.

#### CBO feedback

Likely due to capacity constraints and high demands on CBO time, we received evaluative feedback from only one CBO, restricting a complete evaluation of the PBA model from the CBO perspective. However, the sole CBO PBA respondent reported a very positive experience and the beginning of a longer-term collaborative relationship:

*“[Consultant] is so great!. I know that she’s a person we know we can turn to whenever we need since she told us that. She came to our [annual community celebration] as our guest and was so happy to be there.”*

#### Consultant feedback

Two consultants provided positive general feedback:

*PBA Consultant 1 “I have been learning a lot from this project.”*

*PBA Consultant 2 “I have no complaints about the process and it has been very educational and interesting so far.”*

Two consultants observed that managing time commitments for both themselves and the CBO partners was a learning experience for them:

*PBA Consultant 3 “No challenges, other than [my] time commitment can be difficult at times.”*

*PBA Consultant 4 “Sometimes [CBO partner is] slow to respond when I send them intermediate results for which I need feedback… so we may have to adjust what we are able to produce.*

One consultant suggested a future meeting of all to “present their work/successes/challenges to learn from one another.” Three consultants did not report feedback or suggestions of improvement to the PBA model on evaluation forms.

#### Consultant challenges

Two consultants mentioned difficulties in aligning with CBO expectations. In the words of one consultant:

*PBA Consultant 1 “I reminded them it’s not ‘my survey’ but ‘their survey’ to encourage their leadership and ownership. It was challenging to balance my role as a researcher since they seemed to expect me to construct the survey, potentially reflecting a power dynamic where I was viewed as the expert due to my academic background.*”

## Discussion

We present a no-cost, doctoral student-led innovation that advances NIH’s objective of community-academic partnership by identifying meaningful capacity-building opportunities for CBO and academic partners, including a broad, multidisciplinary spectrum of staff, faculty, post-docs, and students. CRCI offers a framework for bidirectional capacity building between CBOs and the academic community. Leading CRCI is also an opportunity for doctoral students to develop leadership skills and skills in working across community-academic boundaries. Preliminary evidence suggests that the CRCI model is acceptable to academic and community partners alike, given the number and diversity of participants and CBOs who engaged with the initiative. Structured community-academic training and partnership models have been described previously, including the Detroit Urban Research Center’s CBPR Partnership Academy and partnership-readiness and capacity-building approaches developed by Andrews and colleagues (18, 19). CRCI is distinct from these models in its specific configuration: a no-cost, doctoral student-led initiative that pairs a flexible, drop-in Office Hours format with longer-term project-based assistance, draws consultants from a broad multidisciplinary pool across an academic medical system, and is embedded within routine academic responsibilities rather than delivered as a dedicated, funded, year-long curriculum. To our knowledge, this particular combination of features has not been previously reported.

We highlight that participation at Office Hours of community members affiliated with non-CBO entities (e.g., clergy, administrators at other academic medical centers, affiliates of our own department) presented an unexpected opportunity for inter-disciplinary and inter-professional learning between academic staff, CBOs, and other community members and for peer-to-peer training between departmental colleagues.

The CRCI model can be strengthened in a number of ways. For Office Hours, high engagement with CBO staff, as expressed during verbal publicity presentations and in high session registration counts, typically did not equate to high session attendance. While the high pre-session engagement validates the model’s acceptability to CBO partners, this mode of community-academic partnership will benefit from further clarification of best practices in converting pre-session engagement to session attendance. Additionally, the Office Hours model faced challenges in balancing the flexibility needed to address individual needs with advance preparation required by consultants on specific topics, as well as ensuring that CBO staff were ready with questions or discussion topics beforehand. Developing best practices in this area will benefit model refinement.

Implementation of CRCI was a pilot initiative that provides a framework for future student-led efforts and serves as a roadmap demonstrating the model’s feasibility and acceptability. Other students or academic institutions can build upon this model to create an even more impactful and sustainable initiative. We learned that the implementation of Office Hours is quite manageable for CRCI leaders, simultaneously with other academic responsibilities. However, while we did not quantify our own time and efforts in implementing Office Hours or PBA, the demands of PBA’s higher-input facilitation of a longer-term collaborative relationship between academic and community partners require more focused attention and ongoing follow-up with both parties by CRCI leaders. Collectively, these circumstances can be challenging when managing multiple PBA relationships in conjunction with Office Hours and non-CRCI academic obligations. Implementers of similar CRCI models at other sites may benefit from alternating one format at a time (e.g., exclusively offering Office Hours for a given number of months and subsequently managing baseline and follow-up PBA) or dividing management of Office Hours and PBA between smaller teams or groups of CRCI leaders.

Our findings are limited by low response rates to Office Hours and PBA evaluations and short evaluative follow-up periods, particularly for the PBA partnerships. Our lack of PBA evaluation from the perspective of CBO partners further limits our findings. We were challenged to achieve high response rates to PBA evaluations, considering competing demands on consultants, CBOs, and our own professional obligations and varying project timelines across consultant/CBO pairings. In terms of office hours, while sessions were evaluated very favorably by respondents, the positive evaluations may reflect a selection bias. Several additional limitations should be considered when interpreting these findings. First, the generalizability of this work is limited. CRCI was developed and implemented at a single academic medical center within one large urban health system in New York City, drawing on its specific institutional resources, listservs, established Community Advisory Boards, and existing community relationships. The model’s feasibility and acceptability may differ in settings with fewer trainees, smaller community partner networks, or different institutional supports, and our results may not transfer directly to other contexts. Second, the favorable evaluations are subject to selection and response bias. Evaluations were anonymous and voluntary, and only 20 of 90 Office Hours registrations resulted in a completed CBO evaluation, while PBA feedback was received from only one CBO. Respondents who chose to complete evaluations may have been those with more positive experiences or stronger engagement, which could inflate the apparent acceptability of the model; the perspectives of non-respondents, those who registered but did not attend, and partnerships that did not progress are underrepresented. Third, consultants were not required to complete formal training in community-engaged research beyond a brief orientation, which may have affected the consistency and quality of sessions and the experience of CBO partners; future iterations may benefit from a more structured training and competency-development component for consultants. We did not formally quantify these effects or adjust for them, given the program’s quality-improvement design. Future implementation efforts must consider the feasibility of evaluative efforts, particularly of ongoing PBA collaborations, and in the examination of longer-term outcomes associated with both models. Looking ahead, the CRCI pilot concluded with the completion of the founding co-leads’ doctoral training, and we intend this work to function as a feasibility demonstration and roadmap rather than as a continuing program. The materials developed through the initiative, including the consultant orientation, intake form, and standardized MoU template, are documented here and in the Appendix so that future student-led teams, at our institution or elsewhere, can adopt the model and build upon it, including by incorporating the more structured consultant competency-development component identified above. Because CRCI is a no-cost, volunteer-based model embedded within existing academic training structures, replication does not depend on a discrete funding cycle; the model’s durability lies in its transferability rather than in the continuity of any single program or leadership team. In this way, the pilot lays the groundwork for our long-term goal of a multi-site CRCI Network.

In conclusion, we invite the expansion of the reach of CRCI’s model through implementation in any setting engaged in community-academic partnerships. Our long-term goal of a CRCI Network across sites would facilitate rich cross-pollination of ideas and resources relevant to consultants and CBOs alike across CRCI sites.

## Data Availability

The raw data supporting the conclusions of this article will be made available by the authors, without undue reservation.
