# Supplementary material for: Building community-engaged research capacity through bidirectional collaboration: the community research consulting initiative model at NYU Langone health
Source: Front Public Health. 2026 Aug 18;14:1816942. doi: 10.3389/fpubh.2026.1816942 (PMC13530002; doi:10.3389/fpubh.2026.1816942)
Supplement: Supplementary file 1 [file Data_Sheet_1.PDF]

Default Question Block

Community Research Consulting Initiative (CRCI) is a volunteer-based initiative that connects community-based organizations with NYU Langone Health research consultants to facilitate a bidirectional skill share exchange. Research consultants are NYULH faculty, staff, post-docs and graduate students who are volunteering their time to CRCI.

This is a great opportunity for CBO's to receive support from research consultants at no cost and, in turn, provide opportunities to academic partners to gain experience in applied community-based settings.

For more information, contact Deborah Onakomaiya (deborah.onakomaiya@nyulangone.org) and Miriam Gofine (miriam.gofine@nyulangone.org). Deborah and Miriam are second-year doctoral students in Population Health at NYU Grossman School of Medicine.

What is your organization called?

What is your organization's website? *(optional)*

Please provide a description of your organization's mission, services, and/or activities

Name of contact person at your organization

Contact person's email address

CRCI offers two formats for collaboration between Consultants and community organizations. Which of the following formats would meet your organization's needs?

Select all that apply

- ☐ Monthly 1-hour drop in sessions ("Office Hours") on specific topics
- ☐ Project-based assistance (longer-term commitment; single consultant is available for a few hours a month over a few months)

Complete this section if you are interested in <u>monthly 1-hour drop in sessions ("Office Hours")</u>

Monthly 1-hour drop in sessions ("Office Hours") are an opportunity for CBO organizations to receive ad-hoc assistance on a specific topic(s) from 1-2 host Consultants. Sessions will be open to all CBO partners who are involved with CRCI.

If your organization is interested in monthly 1-hour drop in sessions, what topics would be useful to your organization?

For example:
- Analyzing quantitative client data from your organization
- Analyzing transcripts from interviews or focus group

What time of day would work best for you to attend office hours?

- ☐ Morning (9-11)
- ☐ Mid-Day/Lunch (11-2)
- ☐ Late afternoon (2-5)
- ☐ Evening (5-7)

☐ Other

Complete this section if you are interested in longer-term project-based assistance

If your organization is interested in longer-term project-based assistance, which projects at your organization are you seeking assistance with?

Please describe the project(s) in as much detail as possible.

Please consider including:
- Project name
- Timeline
- Learning opportunities for Consultants

CRCI Consultants have many different skills. Some of them may meet your organization's needs. Please choose the skills(s) that match your needs.

This list is not exhaustive - you can request skills not listed below.

- ☐ Quantitative data analysis (e.g., creating statistics from information your organization already collected)
- ☐ Qualitative data analysis (e.g., analyzing transcripts from interviews or focus group)
- ☐ Research design (e.g., thinking through the best way to collect information; structuring a new research project)
- ☐ Survey development (e.g., writing and structuring survey questions and formats)
- ☐ Data collection (e.g., running focus groups, interviews; distributing intake forms)
- ☐ Data management (keeping track of your information using software like Excel, SurveyMonkey, Qualtrics, Google Forms)
- ☐ Using software (e.g., Canva, MailChimp, Social Media)
- ☐ Educational material development (e.g., health-related materials for community members)

- ☐ Program evaluation (e.g., structuring the process for evaluating a program your organization offers)
- ☐ Grant writing
- ☐ Other (enter below)

If you're looking for a Consultant who speaks a language other than English, please list that language(s) here

When does your organization need assistance?

- ☐ Right now
- ☐ April 2022
- ☐ May 2022
- ☐ June 2022
- ☐ July 2022
- ☐ August 2022
- ☐ September 2022 - check back with me then

For project-based assistance, approximately how many consulting hours per week would be requested for your

project(s)?

CRCI is a bidirectional model. In addition to consultants sharing skills with CBO's, they are seeking to learn about applied practices from CBO's.

What skills/experiences could Consultants expect to gain from partnership with your organization?

e.g.: Stakeholder engagement skills; in the field experience, etc.; networking; understanding the types of data that CBO's have

How did you hear about CRCI?

If you already collaborate with anyone at the Department of Population Health at NYU Grossman School of Medicine, please list your colleagues' names here

Anything else you'd like to share?

If you have any additional questions, please feel free to email Deborah Onakomiaya (deborah.onakomaiya@nyulangone.org) and Miriam Gofine (miriam.gofine@nyulangone.org)

Powered by Qualtrics
