# Supplementary material for: Building community-engaged research capacity through bidirectional collaboration: the community research consulting initiative model at NYU Langone health
Source: Front Public Health. 2026 Aug 18;14:1816942. doi: 10.3389/fpubh.2026.1816942 (PMC13530002; doi:10.3389/fpubh.2026.1816942)
Supplement: Supplementary file 2 [file Data_Sheet_2.PDF]

Default Question Block

Name

Email address

Which session(s) are you interested in hosting? We will confirm the session you are assigned to via email.

Wednesday, November 9: 10-11 AM (Grant writing)

Thursday, Dec 1: 12:30-1:30 PM (Data analysis (quantitative))

Tuesday, Dec 13: 3-4 PM (Data collection (e.g., focus groups, interviews, intake forms))

Friday, Jan 13: 10-11 AM (Program evaluation)

Monday, Jan 23: 12:30-1:30 PM (Data analysis (qualitative))

Tuesday, Feb 7: 3-4 PM (Survey development; Data management (e.g., Excel, SurveyMonkey, Qualtrics, Google Form))

None of these times work for me - check in with me next semester

Other

Any other comments?

Powered by Qualtrics
