# Supplementary material for: Building community-engaged research capacity through bidirectional collaboration: the community research consulting initiative model at NYU Langone health
Source: Front Public Health. 2026 Aug 18;14:1816942. doi: 10.3389/fpubh.2026.1816942 (PMC13530002; doi:10.3389/fpubh.2026.1816942)
Supplement: Supplementary file 3 [file Data_Sheet_3.PDF]

All responses are anonymous

Community Research Consulting Initiative: Office Hours Evaluation

We invite your feedback on the Office Hours session that you attended. Your feedback is important to us. Please complete this evaluation so that we can determine how we did. This survey should take not more than 3 minutes to complete. Thank you!

What is your email? (optional)

Which office hours session are you evaluating?

November 9th grant writing
December 1st data analysis (quantitative)
December 13th data collection (focus groups, interviews, Intake forms)
January 13th program evaluation
January 23rd data analysis (qualitative)
February 7th survey development

What populations do you work with?

Black Communities
Latino Communities
Underserved Populations
Vulnerable Populations
Other

I learned something useful from today's consultation

Strongly agree

Agree

Neither agree nor disagree

Disagree

Strongly disagree

The consultant(s) was knowledgeable about the training topic

Strongly agree

Agree

Neither agree nor disagree

Disagree

Strongly disagree

The consultant(s) answered questions adequately

Strongly agree

Agree

Neither agree nor disagree

Disagree

Strongly disagree

The information I received from today's consultation was helpful

Strongly agree

Agree

Neither agree nor disagree

Disagree

Strongly disagree

I am likely to utilize the information I received

Strongly agree

Agree

Neither agree nor disagree

Disagree

Strongly disagree

What was useful about the consultation?

What could be improved?

Suggestion(s) for future office hours topics

OPT OUT HERE: Please check this box if you do not want your email address to be added to CRCI's mailing list to stay in the loop about upcoming events and opportunities *(you can unsubscribe at any time)*

Opt out (list email address below)

Block 1

Powered by Qualtrics
