# Supplementary material for: Building community-engaged research capacity through bidirectional collaboration: the community research consulting initiative model at NYU Langone health
Source: Front Public Health. 2026 Aug 18;14:1816942. doi: 10.3389/fpubh.2026.1816942 (PMC13530002; doi:10.3389/fpubh.2026.1816942)
Supplement: Supplementary file 4 [file Data_Sheet_4.PDF]

The SQUIRE reporting checklist

For checking that reports that describe systematic work to improve the quality, safety and value of healthcare, using a range of methods to establish the association between observed outcomes and interventions. can be understood and used by everyone

How to use this reporting checklist

This reporting checklist allows authors to demonstrate that their manuscripts adhere to the SQUIRE reporting guideline.

If you have not used a reporting guideline before, read about how and why to use them and check whether SQUIRE is the most applicable reporting guideline for your work.

Reporting guidelines are most useful when used early in research. When writing a manuscript or application, consider using the full guidance where you'll find explanations and examples for each item.

After writing, demonstrate adherence by completing this checklist:

1. Specify where each item is described (see Note 1).
2. Cite this checklist (See Note 2).
3. Include your completed checklist as a supplement when submitting to a journal so that future readers can use it to find information.

	Item Description	Location (or reason for not reporting)
**Title and Abstract**		
1. Title	Indicate that the manuscript concerns an initiative to improve healthcare (broadly defined to include the quality, safety, effectiveness, patient-centredness, timeliness, cost, efficiency and equity of healthcare).	Title (p. 1); the title and Abstract identify CRCI as an initiative to improve the equity and capacity of community-engaged healthcare research.
2. Abstract	2a. Provide adequate information to aid in searching and indexing.<br>2b. Summarise all key information from various sections of the text using the abstract format of the intended publication or a structured summary such as: background, local problem, methods, interventions, results, conclusions.	Abstract (p. 2), structured as Objectives, Study Design, Methods, Results, Conclusions.
**Introduction**		

3 & 4. Problem description & Available Knowledge	3. Nature and significance of the local problem.<br>4. Summary of what is currently known about the problem, including relevant previous studies.	Background/Introduction (pp. 3-4); Development of CRCI (pp. 4-6).
5. Rationale	Informal or formal frameworks, models, concepts, and/or theories used to explain the problem, any reasons or assumptions that were used to develop the intervention(s), and reasons why the intervention(s) was expected to work.	Background/Introduction (pp. 3-4); rationale for bidirectional capacity building and the equal-partnership gap, with reference to community-engaged research (CEnR) capacity-building frameworks.
6. Specific aims	Purpose of the project and of this report.	Background/Introduction (end of Introduction, p. 4); Abstract, Objectives (p. 2).
**Methods**		
7. Context	Contextual elements considered important at the outset of introducing the intervention(s).	Methods, Context within Development of CRCI and Consultant/CBO Recruitment (pp. 5-8); single academic medical center within one urban health system, departmental listservs, Community Advisory Boards, and existing community relationships.
8. Intervention(s)	A. Description of the intervention(s) in sufficient detail that others could reproduce it<br>B. Specifics of the team involved in the work.	Methods, Development of CRCI and Office Hours/PBA format definitions (pp. 6-9); team identified as two doctoral-student co-founders with senior faculty advisors (Development of CRCI, p. 5; Author list).
9. Study of the Intervention(s)	A. Approach chosen for assessing the impact of the intervention(s)<br>B. Approach used to establish whether the observed outcomes were due to the intervention(s).	Methods, Study Design and Data Collection (pp. 7-12); formative program evaluation of feasibility, acceptability, and preliminary outcomes.
10. Measures	A. Measures chosen for studying processes and outcomes of the intervention(s), including rationale for choosing them, their operational definitions, and their validity and reliability	Methods, Data Collection and Office Hours/PBA Evaluation (pp. 9-12); Zoom registration logs, post-session Qualtrics surveys (5-point Likert items,

	B. Description of the approach to the ongoing assessment of contextual elements that contributed to t…	full instruments in Appendix), and email follow-ups.
11. Analysis	A. Qualitative and quantitative methods used to draw inferences from the data<br>B. Methods for understanding variation within the data, including the effects of time as a variable.	Methods, Analysis (p. 12); descriptive statistics and basic qualitative content review; data analyzed retrospectively, consistent with the quality-improvement design.
12. Ethical considerations	Ethical aspects of implementing and studying the intervention(s) and how they were addressed, including, but not limited to, formal ethics review and potential conflict(s) of interest.	Methods, PBA Evaluation (pp. 11-12) and Ethical approval statement; determined exempt by the NYU Grossman School of Medicine IRB as a quality-improvement initiative. Conflicts of interest: Declaration of competing interests.
**Results**		
13 a & b. Evolution of the intervention and details of process measures	13a. Initial steps of the intervention(s) and their evolution over time (e.g., time-line diagram, flow chart, or table), including modifications made to the intervention during the project.<br>13b. Details of the process measures and outcome.	Methods, Development of CRCI and Results, Office Hours (pp. 6-7, 10-12); Table 1 (stakeholder input and adaptations), Table 2 (implementation), Figure 1 (flow). Process/outcome measures in Results (pp. 10-13).
13 c, d & e Contextual elements and unexpected consequences	13c. Contextual elements that interacted with the interventions<br>13d. Observed associations between outcomes, interventions and relevant contextual factors<br>13e. Unintended consequences such as benefits, harms, unexpected results, problems or failures associated with the intervention(s)	Results and Discussion (pp. 11-15); registration-to-attendance gap, expectation/format mismatch, unexpected participation by non-CBO attendees, and consultant-reported challenges (time, responsiveness, power dynamics).
13 e. Missing data	13e. Details about missing data.	Methods, Data Collection (p. 9) and Results (pp. 12-13); attendance counts unavailable (sessions not recorded; consultant-reported), low evaluation response (20/90 Office Hours; one CBO PBA evaluation).

**Discussion**		
14. Summary	14a. Key findings, including relevance to the rationale and specific aims<br><br>14b. Particular strengths of the project	Discussion (pp. 14-15); key findings and strengths of the no-cost, student-led bidirectional model.
15. Interpretation	15a. Nature of the association between the intervention(s) and the outcomes<br><br>15b. Comparison of results with findings from other publications<br><br>15c. Impact of the project on people and systems<br><br>15d. Reasons for any differences between observed and anticipated outcomes, including the influence of context<br><br>15e. Costs and strategic trade-offs, including opportunity costs	Discussion (pp. 14-15); interpretation, comparison with prior models (Coombe et al. 2020; Andrews et al. 2011), impact, reasons for differences from anticipated outcomes; no-cost model (cost trade-offs noted).
16. Limitations	16a. Limits to the generalisability of the work.<br><br>16b. Factors that might have limited internal validity such as confounding, bias, or imprecision in the design, methods, measurement, or analysis.<br><br>16c. Efforts made to minimise and adjust for limitations.	Discussion, Limitations (p. 15); generalizability (single site/system), selection and response bias, absence of formal CEnR training, and efforts/constraints noted under the quality-improvement design.
17. Conclusion	17a. Usefulness of the work<br><br>17b. Sustainability<br><br>17c. Potential for spread to other contexts<br><br>17d. Implications for practice and for further study in the field<br><br>17e. Suggested next steps	Discussion/Conclusion (pp. 15-16); usefulness, sustainability, potential spread (proposed CRCI Network), implications for practice and research, and next steps.
**Other information**		
18. Funding	Sources of funding that supported this work. Role, if any, of the funding organisation in the design, implementation, interpretation and reporting.	Funding statement (p. 17); CIHR, NIMHD (1F31MD019216-01), and NYU CTSI (UL1 TR001445). No funder role in design, implementation, interpretation, or reporting.
