# Supplementary material for: Building community-engaged research capacity through bidirectional collaboration: the community research consulting initiative model at NYU Langone health
Source: Front Public Health. 2026 Aug 18;14:1816942. doi: 10.3389/fpubh.2026.1816942 (PMC13530002; doi:10.3389/fpubh.2026.1816942)
Supplement: Supplementary file 5 [file Data_Sheet_5.PDF]

Appendix: CRCI Recruitment, Outreach, and Partnership Materials

Note on confidentiality: Outreach emails reproduced below have been limited to the text used to invite participation and circulate opportunities. Personal contact details of external community partners (individual names, email addresses) and any embedded survey/registration data have been removed. Founder contact information is retained as it appears in the published manuscript. Hyperlink destinations have been replaced with bracketed descriptions.

Appendix A. NYU H+H CTSI Community Advisory Board Memorandum of Understanding (Template)

This is the NYU H+H Clinical & Translational Science Institute (CTSI) Community Advisory Board Memorandum of Understanding template. Bracketed fields [ ] indicate placeholders completed for each partner.

NYU H+H CTSI
MEMORANDUM OF UNDERSTANDING
NYU H+H Clinical & Translational Science Institute and [CBO]
[Dates] Budget Period | [Dates] Project Period
Community Advisory Board Member Site: [CBO]

This memorandum of understanding outlines the formal partnership between the New York University – NYC Health + Hospitals Clinical and Translational Science Institute (NYU H+H CTSI) and [CBO] in carrying out program activities as a NYU H+H CTSI Community Advisory Board Member. These roles and responsibilities are not limited to those stated below. Modifications, when necessary and agreed to by both parties, to better facilitate and implement the goals and activities of the project can be made after reviewing the overall progress.

STATEMENT OF WORK: The NYU H+H CTSI shall work in partnership with [CBO] to support efforts related to: 1) providing guidance on developing and maintaining sustainable community partnerships; 2) serving on the advisory committees and workgroups for the NYU-H+H CTSI; 3) assisting with identifying research priorities; 4) providing input for the development of research and research training programs; and 5) assisting in the dissemination of evidence-based strategies to reduce and eliminate health disparities in New York City.

The group represents a diverse cross-section of New York City's ethnic communities, government, healthcare community, social services, and neighborhoods and boroughs.

[CBO Description]

The collaboration between the NYU H+H CTSI and [CBO] will consist of the following in Year 1 of the project:
[CBO] will participate in quarterly community advisory board (CAB) meetings per year (4 meetings/year), including the following responsibilities:

- Reviewing CTSI Pilot Projects (1x/year, up to 2 proposals)
- Reviewing protocols and providing input on community-engaged research strategies (up to 3 proposals/year)
- Providing feedback and input on fliers, guides, and training manuals related to community-engaged research
- Participation in annual Health Disparities Symposium and promotion of events
- Participation in small collaborative projects (i.e., digital stories)
- Providing letters of support for collaborative grant opportunities

- Opportunity to participate in Community Engagement Studios (additional hourly compensation provided; up to 10 hours per year)

NYU H+H CTSI will include the following responsibilities:

- Provide honorarium and/or small grants for projects
- Full-time Data Analyst supported by CTSI to support evaluation of CBO program data / analysis of secondary data
- Trainings on topics recommended by CAB partners
- Assist in reviewing CBO grant applications
- Facilitate connections to academic partners and NYU faculty for collaborative community engagement activities and speaking engagements
- Provision of letters of support for grant or other applications
- Promotion of CBO events/programs through our networks
- Participation in at least one collaborative grant opportunity with NYU
- Dissemination of CBO program or evaluation findings through publication, report, etc., including working with community partners on co-authoring publications.

Financial Agreement

A payment of $XX will be given to [CBO] for facilitating the activities outlined above. NYU H+H CTSI will request payment from the NYU Finance department for [CBO] after the vendor onboarding process has been completed. Payment will be processed in [#] installment(s). Honorarium payment is contingent on engagement on the CAB.

NYU H+H CTSI CEPHR Co-Directors, CAB co-chairs, and membership will periodically review this memorandum of understanding and may propose amendments. Both representatives from NYU and [CBO] must approve all changes.

All publications that result from the work and effort of the Center grant activities must acknowledge the National Center for Advancing Translational Sciences, National Institutes of Health under award number UL1 TR001445.

______________________________ ______________________________

[NYU Representative] [CBO Representative]

Nadia Islam, PhD – Co-Director, Community Engagement & Population Health Research Program (CEPHR), NYU H+H CTSI

Antoinette Schoenthaler, EdD, FACH – Co-Director, Community Engagement & Population Health Research Program (CEPHR), NYU H+H CTSI

Appendix B. Recruitment and Outreach Emails

These are the email texts used to invite participation and to circulate CRCI opportunities to consultants and to community-based organizations (CBOs). Hyperlinks are indicated in brackets.

B1. Town hall follow-up – consultant recruitment and CBO circulation

Dear DPH Community,

We are contacting you to follow up on today's [Town Hall presentation link] about the Community Research Consulting Initiative. Your participation is invited!

- Consult: Sign up [Qualtrics consultant sign-up link] to volunteer as a CRCI Consultant (please feel free to share this with any staff, faculty, or students in the NYULH community who might be interested).
- Spread the Word: CRCI's upcoming pilot July–August Office Hours flier is attached and below. Please circulate it to CBOs in your personal and professional networks who may be interested. Pre-registration is required (links are on the flier).
- Collaborate: Please feel free to email us with questions, comments, and/or suggestions. We are leveraging community partnership and incorporating community feedback in this process; we are happy to learn more about everyone's thoughts.

Best,

Deborah and Miriam

Deborah Onakomaiya and Miriam Gofine, Co-Founders, Community Research Consulting Initiative | Doctoral Students, Population Health, Department of Population Health, NYU Grossman School of Medicine

B2. Consultant onboarding update – Office Hours pilot and PBA matching

Dear Colleagues,

Thank you for volunteering to participate in the Community Research Consulting Initiative. A few updates:

- Office Hours: The Office Hours 3-month pilot phase is starting! A draft [schedule link] is online and open for sign-up by consultant hosts. We will circulate the schedule to CBOs once topics/hosts/dates/times are confirmed with consultants. Please take a look and choose a date/time/topic that works for you. This is a pilot and a draft schedule: dates and topics are still flexible. Please email us if our proposal doesn't align with your calendar, if you'd like to host a session on a topic that's not represented, or with any feedback. You'll see 2 host sign-up slots per session; while not required, we encourage co-hosting with a colleague, though consultants are welcome to host independently.
- Project-based consulting: We're working on matching consultants with CBOs. If you indicated interest in participating, please expect to hear from us in the next few weeks.
- We're still recruiting Consultants & CBOs! You're invited to circulate the CRCI opportunity to other colleagues and CBOs in your personal and professional networks.

Please don't hesitate to reach out with further questions or suggestions.

Best, Deborah and Miriam

B3. CBO partner recruitment

Dear DPH Community,

The Community Research Consulting Initiative is actively recruiting community-based organization partners who are interested in receiving no-cost support from NYULH research consultants. We invite you to circulate the recruitment information to CBOs within your networks. Please see the flier and link to our CBO intake form below. Spring recruiting of CBO partners ends on Friday, May 6th. Thank you in advance.

Sincerely, Deborah and Miriam

CBO sign-up [CBO intake form link] by Friday, May 6 – NO-COST RESEARCH SUPPORT OPPORTUNITY FOR CBOs

B4. Consultant information-session invitation (with Q&A)

VOLUNTEER OPPORTUNITY

The Community Research Consulting Initiative (CRCI) invites you to an informational session on Tuesday, February 7, 3:00–3:15 (3:15–4:00 Q&A). CRCI is a volunteer-based student initiative that connects community-based organizations with NYU Langone Health student research consultants to facilitate a bidirectional skill-share exchange. This is a great opportunity for students (PhD/post-docs) to share their expertise with the broader community and to gain experience working in applied settings with community organizations. The informational session will help students learn about the initiative and opportunities for involvement. [Zoom link]. Please register your interest in this [survey link], even if you can't attend.

Q: What kind of skills will be useful to participate in CRCI?
A: Data analysis (quantitative, qualitative); study design; survey development; data collection (e.g., focus groups, interviews, intake forms); data management (e.g., Excel, Survey Monkey, Qualtrics, Google Forms); software (e.g., Canva, MailChimp, social media); educational material development; program evaluation; grant writing… or any other skill you'd like to share.

Q: What's the time commitment?
A: Flexible. Two options: (1) Co-hosting a 1-hour office hours (minimum 1/year); (2) Providing project-based assistance (longer-term commitment or a few hours a month for a few months, according to your availability).

Q: What would students gain by participating in CRCI?
A: Student consultants will gain some of the following: learn how Community-Based Organizations operate in applied settings; skills in engaging and maintaining stakeholder relationships; understanding of community-based programs/services and how they're developed; insight into the types of data CBOs generate.

B5. Consultant welcome email

Dear Community Research Consulting Initiative Consultants,

On behalf of myself and Deborah Onakomaiya, CRCI's co-founder, I want to warmly welcome you to CRCI. We are thrilled that you've volunteered to join the team. A quick update on where we are with the initiative:

- Office Hours: We are piloting Office Hours in July and August. Hosting slots are currently full. Expect to hear about signing up for fall Office Hours in August/September.
- Project-based assistance: We will work on matching consultants and CBOs. We'll provide further updates, with the aim of beginning to pilot this phase in the fall.

Please don't hesitate to reach out with any questions or concerns.

Best, Miriam and Deborah

Appendix C. Recruitment and Office Hours Flyers

Flyer content is transcribed below. Original flyers carried the NYU Grossman School of Medicine branding.

C1. About CRCI – No-Cost Research Consulting for CBOs

Community Research Consulting Initiative (CRCI) is a volunteer-based initiative that connects community-based organizations with NYU Langone Health research consultants to facilitate a bidirectional skill exchange. Research consultants are NYULH graduate students, post-docs, staff, and faculty who volunteer their time to CRCI. This is a great opportunity for CBOs to receive support from research consultants at no cost and, in turn, to provide opportunities to academic partners to gain experience in applied community-based settings. CRCI offers office hours and longer-term project-based assistance. Sign-up is ongoing.

C2. Consultant Recruitment – Info Session (Q&A)

CRCI connects community-based organizations with NYU Langone Health student, staff, and faculty volunteer research consultants to facilitate bidirectional capacity building. This is a great opportunity to share your expertise with the broader community and to gain experience working in applied settings with community organizations.

Q: What kind of skills will be useful? A: Data analysis (quantitative, qualitative); study design; survey development; data collection (focus groups, interviews, intake forms); data management (Excel, Survey Monkey, Qualtrics, Google Forms); software (Canva, MailChimp, social media); educational material development; program evaluation; grant writing; or any other skill you'd like to share.

Q: What's the time commitment? A: Flexible — co-hosting a 1-hour office hours (minimum 1/year), or providing project-based assistance (longer-term or a few hours a month for a few months).

Q: What would consultants gain? A: Learning how CBOs operate in applied settings; skills in engaging and maintaining stakeholder relationships.

C3. Office Hours Schedule – Virtual Q&A Office Hours (June–August 2023)

Attendees: please come with your questions ready! Registration was required for each session (all times EST):

- June 5, 10–11 — Grant Writing
- June 21, 12:30–1:30 — Collecting & Managing Your Data
- July 7, 3–4 — Program Evaluation
- July 18, 10–11 — Mixed Methods: Making Sense of Qualitative + Quantitative Data
- July 27, 12:30–1:30 — Literature Reviews (systematically synthesizing information)
- Aug 7, 3–4 — Visualizing Data + Making Infographics
- Aug 16, 12:30–1:30 — Grant Writing

Note: The schedule above illustrates the per-set cadence described in the Methods — single, topic-specific sessions offered approximately every one to two weeks across the block, with no concurrent sessions, and with grant writing offered twice in response to CBO demand.
